# Transcriptomic Analysis Reveals JAK2/MPL-Independent Effects of Calreticulin Mutations in a *C. elegans* Model

**DOI:** 10.3390/cells12010186

**Published:** 2023-01-02

**Authors:** Ana Guijarro-Hernández, Laura Eder-Azanza, Cristina Hurtado, David Navarro-Herrera, Begoña Ezcurra, Francisco Javier Novo, Juan Cabello, José Luis Vizmanos

**Affiliations:** 1 Department of Biochemistry and Genetics, School of Sciences, University of Navarra, 31008 Pamplona, Spain; 2 Center for Biomedical Research of La Rioja (CIBIR), 26006 Logroño, Spain; 3 Navarra Institute for Health Research (IdiSNA), 31008 Pamplona, Spain

**Keywords:** myeloproliferative neoplasms, JAK2, CALR, MPL, *Caenorhabditis elegans*

## Abstract

There is growing evidence that *Ph*-negative myeloproliferative neoplasms (MPNs) are disorders in which multiple molecular mechanisms are significantly disturbed. Since their discovery, *CALR* driver mutations have been demonstrated to trigger pathogenic mechanisms apart from the well-documented activation of JAK2/MPL-related pathways, but the lack of experimental models harboring *CALR* mutations in a JAK2/MPL knockout background has hindered the research on these non-canonical mechanisms. In this study, CRISPR/Cas9 was performed to introduce homozygous patient-like calreticulin mutations in a *C. elegans* model that naturally lacks *JAK2* and *MPL* orthologs. Whole-genome transcriptomic analysis of these worms was conducted, and some of the genes identified to be associated with processes involved in the pathogenesis of MPNs were further validated by qPCR. Some of the transcriptomic alterations corresponded to typically altered genes and processes in cancer and *Ph*-negative MPN patients that are known to be triggered by mutant calreticulin without the intervention of JAK2/MPL. However, interestingly, we have also found altered other processes described in these diseases that had not been directly attributed to calreticulin mutations without the intervention of JAK2 or MPL. Thus, these results point to a new experimental model for the study of the JAK2/MPL-independent mechanisms of mutant calreticulin that induce these biological alterations, which could be useful to study unknown non-canonical effects of the mutant protein. The comparison with a calreticulin null strain revealed that the alteration of all of these processes seems to be a consequence of a loss of function of mutant calreticulin in the worm, except for the dysregulation of Hedgehog signaling and *flh-3*. Further analysis of this model could help to delineate these mechanisms, and the verification of these results in mammalian models may unravel new potential therapeutic targets in MPNs. As far as we know, this is the first time that a *C. elegans* strain with patient-like mutations is proposed as a potential model for leukemia research.

## 1. Introduction

It is known that *Ph*-negative myeloproliferative neoplasms (polycythemia vera, PV, essential thrombocythemia, ET, and primary myelofibrosis, PMF) are rare hematological malignancies characterized by the clonal expansion of mature myeloid cells. Over the last two decades, researchers have provided key insights into the genetic and molecular mechanisms underlying these diseases. It is now well known that *Ph*-negative MPNs share driver mutations in JAK2 (Janus kinase 2), MPL (thrombopoietin receptor), and CALR (calreticulin) that lead to the constitutive activation of JAK2-related signaling pathways (JAK2/STAT, MAPK/ERK, and PI3K/AKT). Unlike JAK2 and MPL, which play a central role in intracellular signaling, CALR is a Ca^2+^-binding chaperone mainly localized in the endoplasmic reticulum (ER). This multifunctional protein is involved in numerous processes, such as the regulation of intracellular Ca^2+^ homeostasis, the cellular response to ER stress (unfolded protein response, UPR), adipocyte differentiation, proliferation, wound healing, apoptosis, and immunogenic cell death [1,2,3]. Somatic mutations of *CALR* consist of deletions or insertions in exon 9 that shift the reading frame by one base pair (+1), mainly a 52 bp deletion or type 1 mutation (c.1902_1143del, CALR^del52^), and a 5 bp insertion or type 2 mutation (c.1154_1155insTTGTC, CALR^ins5^). As a result, mutant CALR shows a novel C-terminus that lacks the ER retention motif (KDEL) and some Ca^2+^-binding sites. In 2016, it was published that mutant CALR is transported to the cellular membrane where it activates MPL in a ligand-independent manner [4]. However, several studies have shown that these alterations can cause more complex disturbances in the cell through non-canonical mechanisms, such as changes in UPR, cytoskeleton, and ribosomal proteins or increased DNA damage, proliferation and immunoevasion [reviewed in 1].

In order to identify some of the aberrant mechanisms of mutant CALR proteins, in this study we performed whole-genome transcriptomic analyses in a *C. elegans* model harboring homozygous patient-like calreticulin mutations. *C. elegans* is a soil nematode of a small size (1 mm) that grows from egg to adult through four larval stages (L1, L2, L3, and L4). It has been widely used as an in vivo experimental model for its numerous benefits, such as transparency, low-cost, invariant number of somatic cells, and ability to freeze and recover, thus, facilitating the preservation of mutant pure strains. Furthermore, many of the human genes and signaling pathways are conserved in this organism but with less components and redundancy. For this reason, it is a suitable and a simple in vivo model to evaluate the pathogenic potential of genetic alterations [5]. Specifically, this organism shows a series of advantages for this study. First, *CALR* has an ortholog in the nematode genome (*crt-1*) [6] and our group has created *C. elegans* strains harboring type 1 and type 2-like *crt-1* mutations by using CRISPR/Cas9. It is of interest to note that, unlike for mice, a *crt-1* null mutant (KJ216 *crt-1*(*jh101*)) does not result in embryonic lethality, but disrupts several processes, including fertility, stress response [7], UPR [8], development [9], and metabolism [10]. Likewise, according to Ortholist2 [11], this organism has no *JAK2* or *MPL* orthologs, so the aberrant mechanisms observed in this model necessarily correspond to JAK2/MPL-independent functions of mutant CRT-1.

## 2. Materials and Methods

### 2.1. C. Elegans Strains and Maintenance

**Figure 1:**
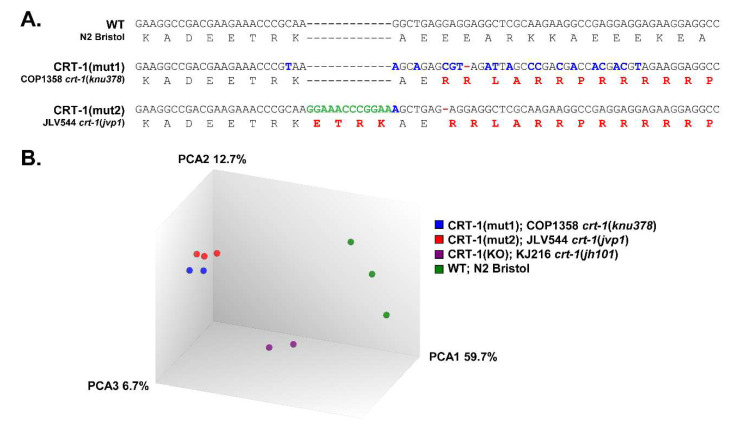
DNA and protein sequences and PCA of wild-type and calreticulin-mutated worms. (**A**) DNA and amino acid sequences of the region of calreticulin that has been mutated by CRISPR/Cas9 to generate changes homologous to those found in patients with MPNs. From top to bottom, as follows: sequence in wild-type (WT) nematodes (N2 Bristol), sequence in CRT-1(mut1) worms (COP1358 *crt-1*(*knu378*)) showing the mutation c.1128del, and sequence in CRT-1(mut2) worms (JLV544 *crt-1*(*jvp1*)) showing the mutations c.1118_1119insGGAAACCCGGAA and c.1125del. In both type 1 and type 2-like mutant strains, the mutations resulted in a +1 bp shift in the reading frame, generating a novel C-terminus with similar properties to that of the mutated CALR found in MPN patients. Silent point mutations (blue) in mutant strains were created to eliminate possible PAM sequences for the CRISPR/Cas9 mutagenesis and to facilitate their detection by molecular methods. Changes in amino acid sequence are shown in red. (**B**) Principal component analysis (PCA) on microarray data. The PCA was performed using the Affymetrix^®^ Transcriptome Analysis Console (TAC) 4.0. The WT control samples (N2 Bristol) are shown as green spheres; CRT-1(mut1) samples (COP1358 *crt-1*(*knu378*)) are shown in blue; CRT-1(mut2) samples (JLV544 *crt-1*(*jvp1*)) are shown in red; CRT-1(KO) samples (KJ216 *crt-1*(*jh101*)) are shown in purple.

Nematodes were maintained at 20 °C on NGM agar plates seeded with the *E. coli* strain OP50, according to standard protocols. The following strains were used: Bristol N2 (wild type; WT), COP1358 (type 1-like *crt-1* homozygous mutant; *crt-1*(*knu378*), hereinafter referred to as CRT-1(mut1)), JLV544 (type 2-like *crt-1* homozygous mutant; *crt-1(jvp1),* referred to as CRT-1(mut2)), and KJ216 (*crt-1* null mutant; *crt-1*(*jh101*) [7], hereinafter referred to as CRT-1(KO)). Bristol N2 and KJ216 were acquired from the *Caenorhabditis* Genetics Center (CGC, University of Minnesota, Minneapolis, MN) while type 1 and type 2-like *crt-1* mutants were obtained using CRISPR/Cas9 technology (Figure 1A). We used computational methods to verify that the proteins generated in the worm maintained the properties previously observed for the human mutant proteins (Table S1) [12].

### 2.2. Characterization of the Larval Development of C. Elegans Strains

Animals were age-synchronized by standard hypochlorite treatment of gravid nematodes to obtain eggs that were allowed to hatch in M9 medium within 48 h. Approximately 400 age-synchronized L1 larvae per strain were transferred onto 2 NGM plates and incubated at 20 °C for 4 days. The length of 25 worms per plate and strain was analyzed at 0, 5, 24, 29, 48, 53, 72, and 75 h using the NIS Elements Documentation software v4.00 and a SMZ18 stereomicroscope equipped with a DS-Fi2 camera (Nikon Instruments Inc., Tokyo, Japan). The time when the strains reached each life stage (L1, L2, L3, early L4, late L4, young adult, or adult) was further identified by both length measurements and the characteristic phenotypic traits of each stage. Finally, a growth curve modeling was performed (data not shown).

### 2.3. RNA Extraction

Approximately 4000 age-synchronized L1 nematodes per strain were grown in triplicate on a total of 8 NGM plates (~500 nematodes per plate) at 20 °C until they reached the L4 stage. Wild-type worms were collected and washed after 44 h, while type 1/type 2-like calreticulin mutants and the *crt-1* null strain were collected after 51 and 56 h, respectively, since they showed a delayed development. We verified that at those time points all the worms were at exactly the same L4 stage, given their length, body size, and the presence of a half-moon of the same size in the central region of their bodies (Figure S1). Total RNA was extracted using TRIzol^®^ Reagent (Thermo Fisher Scientific Inc., Paisley, UK) according to the manufacturer specifications. The RNA was purified from TRIzol^®^ using the Qiagen RNeasy^®^ Mini Kit (Qiagen, Hilden, Germany), following the recommended protocol for RNA cleanup. Concentration and purity of RNA were quantified at 260/280 nm with a NanoDrop ND-100 spectrophotometer (Thermo Fisher Scientific Inc., Wilmington, DE, USA).

### 2.4. Whole-Genome Transcriptomic Analysis

The transcriptomic analysis of RNA samples was conducted by the Cancer Research Center (CIC-IBMCC, University of Salamanca, Salamanca, Spain). Initially, the quality of total RNA was analyzed with the 2100 Bioanalyzer (Agilent Technologies, Santa Clara, CA, USA) using the RNA 6000 Nano Kit. A total amount of 100 ng of RNA was used as a template to obtain biotinylated single-stranded DNA from the entire mRNA using the GeneChip WT PLUS Reagent kit according to the instructions of the manufacturer. Fragmented cDNA preparations were hybridized to the genome oligonucleotide array *C. elegans* Gene 1.0 ST (Thermo Fisher Scientific-Affymetrix, Santa Clara, CA, USA) with the GeneChip Hybridization Oven 645, washed, and then subsequently scanned on a GeneChip Scanner 3000 TG System. Microarray data were normalized with the RMA algorithm (Robust Multiarray Analysis) and analyzed using the Affymetrix^®^ Transcriptome Analysis Console (TAC) 4.0. We considered as differentially expressed all the transcripts with a fold-change (FC) ≤ 2 or ≥ 2 and a false discovery rate (FDR) (*q*-value) < 0.05. Gene set enrichment analysis (GSEA) of gene expression data was performed using the clusterProfiler R package [13] on MSigDB databases for *C. elegans* obtained with the R package msigdbr [14].

### 2.5. Quantitative Real-Time PCR (qPCR)

Here, RT-qPCR validation was performed using both the same RNA samples used for microarray experiments (technical validation) and using independent RNA samples extracted from worms grown at a different time point (experimental validation). The RNA samples from these two biological replicates were reverse transcribed with Invitrogen^TM^ M-MLV Reverse Transcriptase and random primers (Thermo Fisher Scientific Inc., Paisley, UK) according to the standard protocol. Relative mRNA expression of selected genes was validated using qPCR. All reactions were run in 384-well plates using a C100 Touch^TM^ Thermal Cycler CFX384^TM^ Real-Time System (Bio-Rad Laboratories, Hercules, CA, USA). Cycling conditions were as follows: 50 °C for 2 min, 95 °C for 10 min and 40 cycles at 95 °C for 15 s, and 60 °C for 1 min. The reaction mix had a final volume of 10 µL per well composed of 5 µL of iTaq^TM^ Universal Probes Supermix (Bio-Rad Laboratories, Hercules, CA, USA), 0.5 µL of primers and Taqman^®^ probes (20X) (IDT, Integrated DNA Technologies Inc., Coralville, IA, USA), 1 µL of diluted cDNA (12.5 ng/µL) and 3.5 µL of nuclease-free H_2_O. Primers and probes (Table S2) were designed using the PrimerQuest^®^ Tool from IDT (http://eu.idtdna.com/pages/tools/primerquest). Each sample was analyzed in triplicate, and appropriate negative controls were included. The C_T_ (threshold cycle) values were collected using the CFX Manager c3.1 software (Bio-Rad Laboratories, Hercules, CA, USA). The expression level of each gene was normalized to the expression of the housekeeping gene *tba-1* (Applied Biosystems by Thermo Fisher Scientific Inc., Foster City, CA, USA). Gene expression differences between samples were quantified following the 2^-∆∆Ct^ method.

### 2.6. Statistics

Statistical calculations were performed using StataSE v12 software (StataCorp LP, College Station, TX, USA) and GraphPad Prism 8.0.2 (GraphPad Software, San Diego, CA, USA). The significance level (α) was set at 0.05. In this sense, differences were considered as non-significant (NS) when *p* > 0.05, significant (*) when *p* < 0.05, very significant (**) when *p* < 0.01, and highly significant (***) when *p* < 0.001. To compare microarray and qPCR results, a Bland–Altman plot analysis was performed (Figure S2). For qPCR, ∆C_T_ values for each gene were compared between strains using the parametric one-way ANOVA test followed by multiple comparisons.

## 3. Results

### 3.1. Type 1 and Type 2-like Calreticulin Mutants Behave Similarly to Each Other and Differently to the Wild-Type and Knockout Strains and Show a Transcriptional Alteration of Genes Participating in Processes That Have a Role in Cancer and MPNs

The principal component analysis (PCA) on the microarray data shows that type 1 and type 2-like calreticulin-mutated worms cluster together and are clearly separated from wild-type samples and *crt-1* null mutants (Figure 1B). According to the PCA results, type 1-like and null mutants were analyzed in duplicate since one of the samples was aberrant (data not shown).

In order to study the differences in gene expression triggered by mutant calreticulin, we compared the gene expression profile of type 1 and type 2-like calreticulin-mutated nematodes vs. non-mutated samples. We found 1873 differentially expressed genes (DEGs) (863 upregulated and 1010 downregulated) when type 1-like mutants were compared with non-mutated controls and 1993 DEGs (863 upregulated and 1130 downregulated) when comparing type 2-like mutants with non-mutated samples (Tables S3 and S4). Interestingly, type 1 and type 2-like calreticulin mutants behaved differently to the knockout strain. A total of 403 and 488 DEGs were found when comparing type 1 and type 2-like calreticulin-mutated worms to the knockout strain, respectively (Tables S5 and S6). All microarray data were submitted to the Gene Expression Omnibus repository (GEO; http://www.ncbi.nlm.nih.gov/geo, GSE201599).

**Figure 2:**
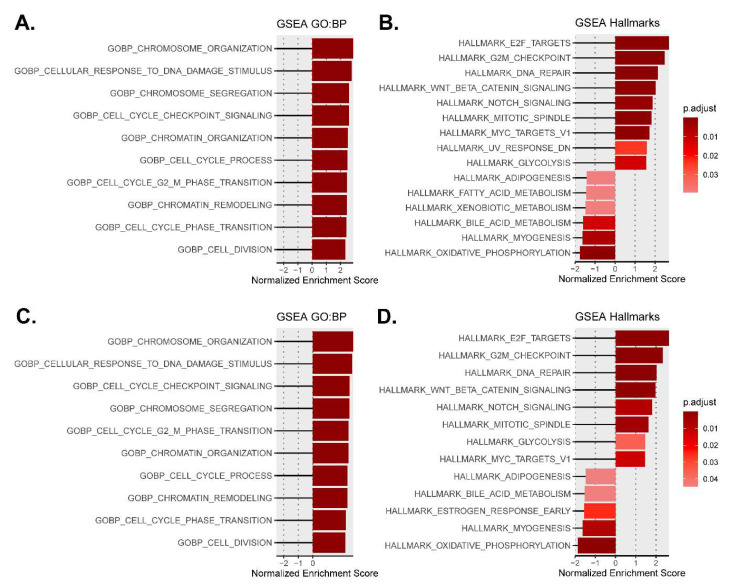
Most altered biological processes and hallmarks in type 1 and type 2-like calreticulin-mutated worms using the normalized enrichment scores from GSEA. (**A**,**B**) Data from CRT-1(mut1). (**C**,**D**) Data from CRT-1(mut2). A darker red color is indicative of lower GSEA adjusted *p*-value. The adjusted *p*-values for all the shown GO biological processes in type 1/type 2-like mutants were < 10^−8^.

**Figure 3:**
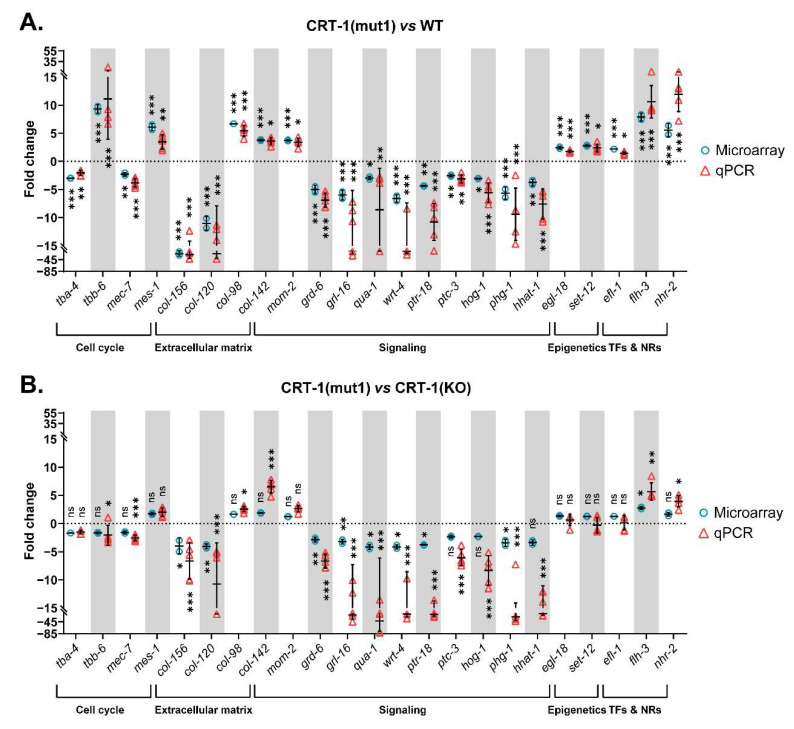
Relative expression levels of genes validated by qPCR classified according to the biological process in which they participate. (**A**) Data from CRT-1(mut1) vs. WT. (**B**) Data from CRT-1(mut1) vs. CRT-1(KO). Blue circles correspond to microarray results, and red triangles represent the qPCR values of two biological replicates. Individual values, means, and SDs are represented. Differences were considered as significant (*) when *p* < 0.05, very significant (**) when *p* < 0.01, and highly significant (***) when *p* < 0.001.

**Figure 4:**
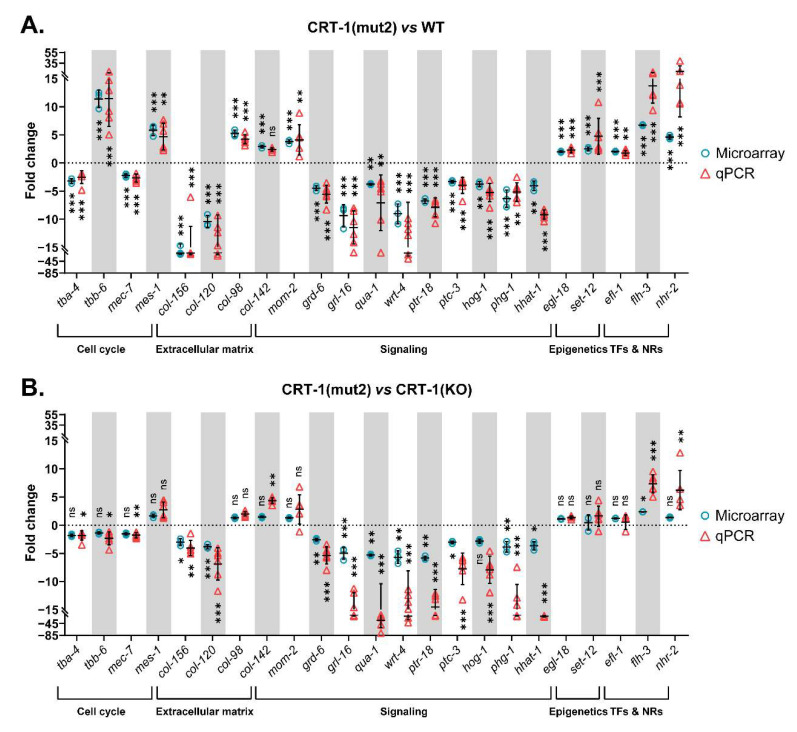
Relative expression levels of genes validated by qPCR classified according to the biological process in which they participate. (**A**) Data from CRT-1(mut2) vs. WT. (**B**) Data from CRT-1(mut2) vs. CRT-1(KO). Blue circles correspond to microarray results and red triangles represent the qPCR values of two biological replicates. Individual values, means, and SDs are represented. Differences were considered as significant (*) when *p* < 0.05, very significant (**) when *p* < 0.01, and highly significant (***) when *p* < 0.001.

The GSEA revealed that the most altered biological processes and hallmarks in both type 1 and type 2-like calreticulin-mutated nematodes (Table S7) were distinguishing signatures of cancer [15] that participate in the pathogenesis of MPNs, such as the activation of the cell cycle, DNA repair mechanisms, and Wnt and Notch signaling, or the dysregulation of cellular metabolism (Figure 2). We also found other features of MPNs to be altered in mutant nematodes, such as the disruption of extracellular matrix and Hedgehog and RTK/Ras/MAPK signaling or an aberrant epigenetics regulation. The expression of some of the most perturbed genes that participate in these processes and have a role in MPNs was validated by qPCR (Figure 3 and Figure 4), showing a strong correlation with the microarray results (Figure S2).

### 3.2. Mutant Calreticulin Induces an Aberrant Expression of Cell Cycle Players

**Figure 5:**
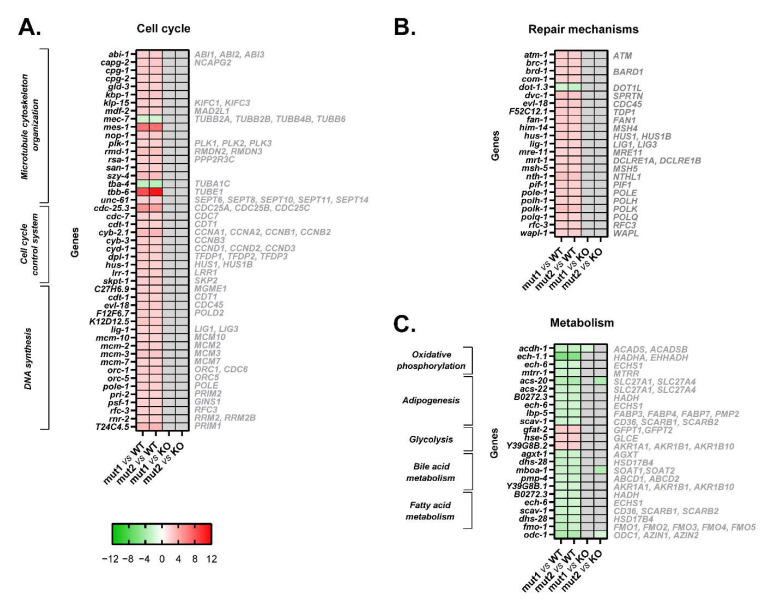
Heatmaps of microarray data showing DEGs related to cell cycle, repair mechanisms, and cellular metabolism in CRT-1(mut1) and CRT-1(mut2) worms vs. WT or CRT-1(KO) nematodes. (**A**) Cell cycle (microtubule cytoskeleton organization, cell cycle control system, and DNA synthesis). (**B**) Repair mechanisms. (**C**) Cellular metabolism (oxidative phosphorylation, adipogenesis, glycolysis, bile acid, and fatty acid metabolism). Mean fold-change values are represented, ranging from shades of green (negative fold-change) to shades of red (positive fold-change). Non-statistically significant differences are shown in gray. Names in gray on the right are the human orthologous genes according to Ortholist2 [11].

As regards the expression of genes involved in the cell cycle, a total of 46 DEGs were found, of which only 2 were downregulated. The products of these genes perform various functions, including microtubule cytoskeleton organization, cell cycle control, and DNA synthesis (Figure 5A).

Among the DEGs related to microtubule cytoskeleton organization, it is of interest to note that type 1 and type 2-like calreticulin-mutated worms showed an altered expression of several tubulins, such as *tba-4* (TuBulin, Alpha), *tbb-6* (TuBulin, Beta), and *mec-7* (tubulin beta-1 chain) (Figure 3A, Figure 4A and Figure 5A). Of these, only *tbb-6* was upregulated. The high upregulation found in *mes-1* (Figure 3A, Figure 4A and Figure 5A), which encodes a protein that seems to directly position the developing mitotic spindle and has an important role in embryogenesis, is also noteworthy [16].

Several cell cycle kinases, phosphatases, cyclins, and other genes involved in cell cycle control were upregulated in type 1 and type 2-like calreticulin-mutated worms, as well as other genes that participate in DNA synthesis, such as those encoding DNA polymerases, ligases, helicases, or primases. Particular attention is drawn to the aberrant expression of seven target genes of MYC in type 1 and type 2-like calreticulin-mutated worms (Figure S3A), since MYC regulates many critical genes for cell proliferation.

In summary, both types of mutations in calreticulin seem to induce an aberrant expression of key cell cycle players without JAK2 or MPL intervention in the worm.

The comparison of the expression of these genes between the type 1/type 2-like mutants and the *crt-1* null strain revealed no differences according to microarray results (Figure 5A). Although some differences were found through qPCR analysis (Figure 3B and Figure 4B), the expression of these genes in type 1/type 2-like mutants was intermediate between the wild-type and the *crt-1* null strain, suggesting that the alteration of the cell cycle observed in type 1 and type 2-like mutants could be a consequence of at least a partial loss of function of the mutant calreticulin in the worm.

### 3.3. Mutant Calreticulin Induces the Activation of Repair Mechanisms

Type 1 and type 2-like calreticulin-mutated worms displayed an increased expression of some genes whose products participate in DNA repair mechanisms. In our model, we found 23 DEGs involved in this process that were upregulated, such as *atm-1* (worm ortholog of human *ATM*), *brd-1* (*BARD1*), *brc-1* (*BRCA1*), *rfc-3* (*RFC3*), and several members of the *MSH* (*him-14, msh-5*) gene families (Figure 5B).

In short, mutant calreticulin might also activate repair mechanisms in a JAK2/MPL-independent manner in the worm.

The expression of these genes involved in repair mechanisms was similar between the type 1/type 2-like mutants and the *crt-1* null strain according to the microarray results (Figure 5B), suggesting that the alteration of repair mechanisms observed in type 1 and type 2-like mutants could also be a consequence of a loss of function of the mutant calreticulin in the worm.

### 3.4. Mutant Calreticulin Dysregulates Cellular Metabolism

The MPN-like calreticulin-mutated worms showed an aberrant expression of 19 genes participating in various metabolic processes, such as oxidative phosphorylation, adipogenesis, glycolysis, bile acid metabolism, and fatty acid metabolism (Figure 5C). All these processes were repressed, except for glycolysis (Figure 2).

Thus, mutant calreticulin seems to trigger some metabolic alterations also without JAK2 or MPL intervention in the worm. The dysregulation of cellular metabolism also seems to be a consequence of a loss of function of the mutant calreticulin in the worm since no major differences have been found in the expression of these metabolic genes between the type 1/type 2-like mutants and the *crt-1* null strain (Figure 5C).

### 3.5. The Introduction of Both Type 1 and Type 2-like Mutations in Calreticulin Seems to Disrupt the Composition of the Extracellular Matrix

**Figure 6:**
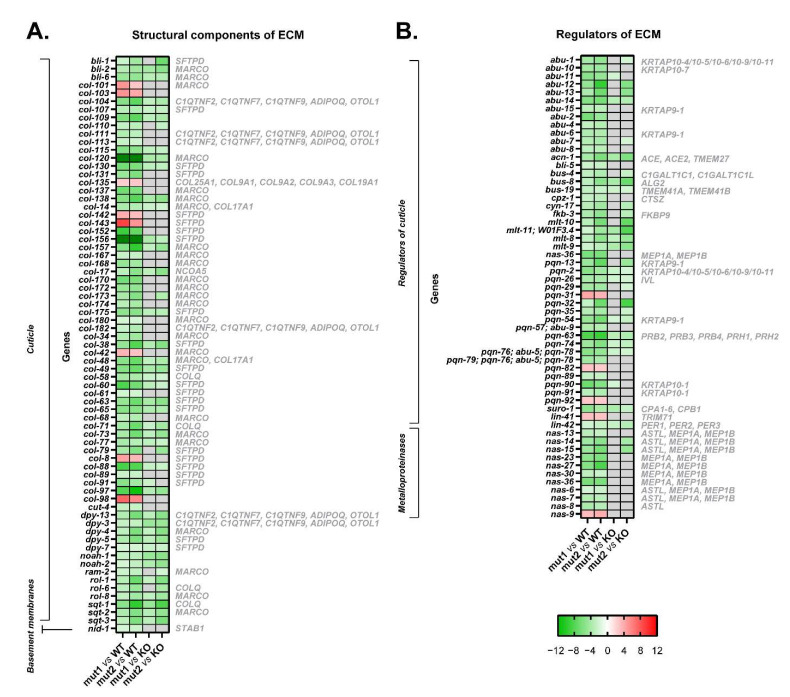
Heatmaps of microarray data showing DEGs related to the extracellular matrix in CRT-1(mut1) and CRT-1(mut2) worms vs. WT or CRT-1(KO) nematodes. (**A**) Structural components of the extracellular matrix (cuticle and basement membranes). (**B**) Regulators of the extracellular matrix (regulators of cuticle and metalloproteinases). Mean fold-change values are represented, ranging from shades of green (negative fold-change) to shades of red (positive fold-change). Non-statistically significant differences are shown in gray. Names in gray on the right are the human orthologous genes according to Ortholist2 [11].

According to gene expression data, the extracellular matrix of worms harboring type 1 and type 2-like calreticulin mutations was disrupted. A total of 70 DEGs in these mutated-calreticulin worms were key components of the cuticle, the exoskeleton of *C. elegans* (Figure 6A). The maximally altered genes, such as *col-156* and *col-120*, encode for collagen proteins and were downregulated (Figure 3A, Figure 4A and Figure 6A). These worms also showed increased levels of some other collagen genes (i.e., *col-98* and *col-142*) (Figure 3A, Figure 4A and Figure 6A) and displayed an aberrant expression of a component of basement membranes (*nid-1*), which are thin layers of a specialized extracellular matrix (Figure 6A).

Additionally, 46 genes involved in the regulation of the cuticle showed an altered expression pattern, and 11 metalloproteinases involved in the proteolysis of the extracellular matrix were also downregulated in calreticulin-mutated worms (Figure 6B).

In short, the introduction of MPN-like mutations in calreticulin also seems to directly disrupt the extracellular matrix of nematodes independently of the JAK2/MPL axis.

Interestingly, the expression of many structural components and regulators of the extracellular matrix was different between the type 1/type 2-like calreticulin mutants and the *crt-1* null strain (Figure 3B, Figure 4B and Figure 6). However, the expression levels of these genes in type 1/type 2-like mutants were intermediate between the wild-type and the knockout strain, suggesting that it could also be a consequence of a partial loss of function of the mutant calreticulin in the worm.

### 3.6. Mutant Calreticulin Worms Show a Different Expression Pattern of Genes Whose Products Participate in Signal Transduction Pathways

**Figure 7:**
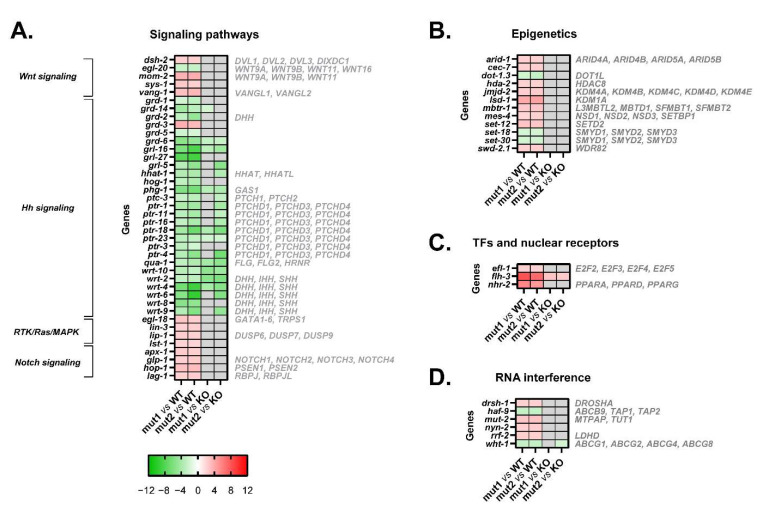
Heatmaps of microarray data showing DEGs related to a set of biological processes in CRT-1(mut1) and CRT-1(mut2) worms vs. WT or CRT-1(KO) nematodes. (**A**) Signaling pathways (Wnt, Hedgehog, RTK/Ras/MAPK, and Notch signaling). (**B**) Epigenetics. (**C**) Transcription factors (TFs) and nuclear receptors. (**D**) RNA interference. Mean fold-change values are represented, ranging from shades of green (negative fold-change) to shades of red (positive fold-change). Non-statistically significant differences are shown in gray. Names in gray on the right are the human orthologous genes according to Ortholist2 [11].

The functional analysis of dysregulated transcripts unveiled differentially activated pathways between type 1/type 2-like calreticulin-mutated and non-mutated worms, such as Wnt, Hedgehog, Notch, and RTK/Ras/MAPK signaling (Figure 7A).

The Wnt signaling seems to be abnormally activated in these calreticulin-mutated worms considering the upregulation of some Wnt ligands (*mom-2*) (Figure 3A, Figure 4A and Figure 7A) and Wnt/β-catenin pathway components (i.e., *dsh-2* and *sys-1*) (Figure 7A).

Similarly, Notch signaling also appears to be more activated in calreticulin-mutant nematodes. In fact, all the DEGs involved in this pathway were upregulated (*glp-1*, *apx-1*, *hop-1*, and *lag-1*) (Figure 7A).

Interestingly, we found an aberrant expression of numerous signal transducers and receptors involved in Hedgehog (Hh) signaling (*grd, grl, qua, wrt,* and *ptr* genes, *ptc-3*, *hog-1*, *phg-1,* and *hhat-1*) most of which were downregulated in calreticulin-mutated worms (Figure 3A, Figure 4A and Figure 7A).

Finally, an abnormal expression of four core components and regulators of the RTK/Ras/MAPK pathway was observed in calreticulin-mutated worms (Figure 7A). Among them, the expression of *egl-18* was validated by qPCR (Figure 3A and Figure 4A).

In conclusion, calreticulin mutations seem to trigger the aberrant expression of genes whose products participate in several signaling pathways by JAK2/MPL-independent mechanisms in the worm. More interestingly, not only because the expression of the genes involved in this pathway was different between the type 1/type 2-like calreticulin-mutated worms and the *crt-1* null strain (Figure 3B, Figure 4B and Figure 7B), but also because the expression of these genes was opposite between patient-like mutants and knockout worms (Figure S4), the dysregulation of Hedgehog signaling seems to be a consequence of a neomorphic function of the mutant calreticulin in the worm.

### 3.7. Mutant Calreticulin also Seems to Disrupt Epigenetic Mechanisms

Interestingly, calreticulin-mutated nematodes showed an increased expression of 12 genes encoding epigenetic regulators (Figure 7B). Among these, we highlighted the upregulation of *set-12* (*SETD2*, *NSD1*, NSD2) (Figure 3A, Figure 4A and Figure 7B). The expression of these genes was similar between the type 1/type 2-like mutants and the *crt-1* null strain according to the microarray results (Figure 7B), suggesting that these alterations observed in type 1 and type 2-like mutants could be a consequence of a loss of function of the mutant calreticulin in the worm.

### 3.8. Transcription Factors Could Shed Some Light on the Aberrant Mechanisms by Which Mutant Calreticulin Alters the Transcriptome

According to GSEA hallmarks, 19 of the genes that are under the control of the EF2-like transcription factors (*efl* family) were DEGs identified in calreticulin-mutated worms (Figure S3B). Of interest, *efl-1* was upregulated in nematodes harboring calreticulin mutations (Figure 3A, Figure 4A and Figure 7C). Our calreticulin-mutated model showed an abnormal expression of genes involved in RNA interference and specifically in miRNA machinery, such as *drsh-1* (*DROSHA*) (Figure 7D). In this sense, the most upregulated transcription regulator in calreticulin-mutated worms, the FLYWCH zinc finger transcription factor homolog *flh-3* (Figure 3A, Figure 4A and Figure 7C), binds to the promoters of several *C. elegans* miRNA genes [17]. In addition, 6 out of the 12 DEGs found between type 1 and type 2-like mutants were ncRNAs (data not shown).

Noteworthily, *nhr-2* (*PPARA, PPARD,* and *PPARG,* among others) was considerably upregulated in nematodes with calreticulin mutations (Figure 3A, Figure 4A and Figure 7C). The peroxisome proliferator-activated receptors are ligand-activated transcription factors of the nuclear receptor superfamily. These receptors activate numerous target genes, thus, regulating a large variety of physiological processes [18].

All the genes discussed in this section showed a similar expression between the type 1/type 2-like mutants and the *crt-1* null strain, except for *flh-3*, whose expression was opposite between patient-like mutants and knockout worms (Figure S4). Thus, the upregulation of *flh-3* seems to be a distinctive new or neomorphic function of the mutant calreticulin in the worm.

## 4. Discussion

Somatic mutations in *CALR* have been found in *Ph*-negative MPN patients and are known to trigger the constitutive activation of JAK2-related pathways. More concretely, mutant CALR is transported to the cellular membrane where it activates MPL in a ligand-independent manner [4]. However, further studies have shown that these alterations can cause more complex disturbances in the cell through non-canonical mechanisms [reviewed in 1]. These mechanisms could be important in the development of the clinical phenotype or in the success, failure, or resistance to treatments directed against the main activation pathways. Some of these mechanisms might be elusive or difficult to detect due to the potent activation of JAK2-related pathways in patient samples with heterozygous *CALR* mutations, or in mammalian models. In this sense, the transcriptomic analysis of a *C. elegans* model harboring homozygous type 1 and type 2-like calreticulin mutations could shed light on the mechanisms derived from mutant calreticulin alone without JAK2 or MPL intervention, since these nematodes do not have an ortholog of *JAK2* or *MPL*. As far as we know, no similar mammalian models with *Calr* mutations in a *Jak2*/*Mpl* knockout background have been described to date. Despite the limitations of *C. elegans* as a model to recapitulate a blood disease, according to the results shown here, this organism seems to be a potential model for the analysis of some of the aberrant mechanisms triggered by mutant calreticulin.

As expected, the transcriptomic analysis of homozygous calreticulin-mutated worms does not show an abnormal expression of all the genes that have a role in MPNs, since mutant calreticulin performs its main pathological role through JAK/STAT activation, but it shows that mutant calreticulin alone can trigger a transcriptional alteration of some processes that have a role in cancer and *Ph*-negative MPNs (cell cycle, DNA repair mechanisms, cellular metabolism, extracellular matrix, epigenetics, and Wnt, Hedgehog, Notch, and RTK/Ras/MAPK signaling) in the worm without the intervention of JAK2 or MPL.

More concretely, the alteration of several tubulins and other genes involved in microtubule cytoskeleton organization in calreticulin-mutated worms is of interest, since the centrosome, the main microtubule organizing center of animal cells, has been extensively related to MPNs [19,20]. The disruption of MYC targets is also noteworthy, since MYC impairs myeloid differentiation and promotes the proliferation and survival of hematopoietic stem cells and multipotent progenitors to drive myeloproliferative neoplasms [21]. Additionally, some of the DEGs involved in repair mechanisms that were upregulated have been described as altered in MPN patients, such as *ATM* (human ortholog of worm *atm-1*), *BARD1* (*brd-1*), *BRCA1* (*brc-1*), *RFC3* (*rfc-3*), and several members of the *MSH* (*him-14*, *msh-5*) gene families [22,23].

It is worth emphasizing that various metabolic processes (oxidative phosphorylation, adipogenesis, bile acid metabolism, and fatty acid metabolism) were repressed in the worms with calreticulin mutations. In line with our results, MPN mice show severe loss of adipose deposits [24]. With respect to bile acid metabolism, a recent study has demonstrated that it is one of the main deregulated metabolic pathways in PMF and ET patients [25]. Finally, the fatty acid composition of platelet membranes is altered in patients with thrombocytosis due to myeloproliferative disorders [26].

Furthermore, the Hh signaling pathway has been shown to play a role in normal hematopoiesis and in the tumorigenesis of hematological malignancies [27]. In this sense, Ptch2 has been demonstrated to drive myeloproliferation and MPN progression [28]. It is of particular interest to note that recent studies have demonstrated that calreticulin mutations activate essential MAPK signaling [29]. Likewise, constitutive MAPK activation in hematopoietic stem cells induces a myeloproliferative disorder [30].

Among epigenetic regulators, we highlighted the upregulation of *set-12* (*SETD2*, *NSD1*, *NSD2*) in type 1 and type 2-like calreticulin-mutated worms. The *SETD2* encodes a histone lysine methyltransferase that seems to have a role in blood malignancies [31] and more specifically in MPNs [22]. Regarding *NSD* genes, a NUP98:NSD1 fusion has been demonstrated to drive MDS/MPN in children [32].

Finally, E2F transcription regulators have been found to be altered in hematological malignancies [33]. Interestingly, *PPARD* has been demonstrated to be upregulated in ET patients [34]. In the case of *PPARG*, it has been extensively linked to MPNs, since its ligands promote the resolution of myelofibrosis in preclinical models [35] and seem to be crucial for the development of new therapeutic approaches for hematological malignancies [36].

Furthermore, both type 1 and type 2-like mutations show a similar functional impact on all the mentioned biological processes, so the differences observed in type 1 and type 2-mutated patients could be more related to their canonical effects through MPL activation than to JAK2/MPL-independent mechanisms.

In support of our results, the aberrant activation of some of these mechanisms by mutant calreticulin has been already pointed out. For example, *CALR* mutations have been demonstrated to activate essential MAPK signaling [29], proliferation, and DNA damage [1]. However, although all the other altered processes observed in mutant worms (cellular metabolism, extracellular matrix, epigenetics, and Wnt, Hedgehog, and Notch signaling) are known to regulate and guide hematopoietic development, they have not previously been described as a direct consequence of mutant calreticulin independently of its action via JAK2 or MPL in MPN patients. This does not imply that mutant calreticulin could have these effects exclusively without the intervention of JAK2/MPL, but that it could also cause these effects without the activation of both players.

Non-mutated calreticulin has an important role in extracellular matrix and collagen secretion and processing, so it is not surprising that the extracellular matrix disruption observed in calreticulin-mutated nematodes may be a consequence of loss of function of the mutant calreticulin [37]. In fact, we have shown that many of the aberrations observed could be the result of a loss of function of the mutant calreticulin (cell cycle, DNA repair mechanisms, cellular metabolism, extracellular matrix, epigenetics, and Wnt, Notch, and RTK/Ras/MAPK signaling). However, it is also interesting to note that the type 1 and type 2-like calreticulin-mutated worms do not behave similarly to the *crt-1* null strain in all cases. Thus, for example, the dysregulation of Hedgehog signaling seems to be a consequence of a neomorphic function of the mutant calreticulin in the worm, as well as the increased expression of *flh-3*. This gene encodes a transcription factor involved in the regulation of the expression of miRNA genes. In any case, both effects of the mutant calreticulin could be of interest, since the mutant protein in humans shows gains and losses of function [38].

Although the alteration of some of the mentioned transcription factors could provide clues about the mechanisms by which mutant calreticulin triggers the features described here, the manner in which the introduction of type 1 and type 2-like mutations lead to these effects remains unknown. A deeper functional analysis of this model could unravel the precise non-canonical mechanisms of mutant calreticulin in the worm, which should be further validated in mammalian models and human cell lines. If these results were validated, it is of interest to note that the JAK2/MPL-independent alterations triggered by mutant calreticulin in our worm model do not seem to be responsible for the initiation of the disease, since the introduction of patient-like calreticulin mutations in in vivo models that lack or have a decreased expression of MPL are unable to induce an MPN [39,40]. However, these alterations may help the development or progression of the disease, and they could be considered as therapeutic targets for patients in whom JAK/STAT inhibition is not yielding the expected results. In fact, it could be convenient to combine JAK/STAT-blocking drugs with others focused on blocking some of these non-canonical mechanisms. The synergies and interactions between them could result in a better and more effective treatment for patients with *CALR*-mutated MPNs.

## 5. Conclusions

To date, research on the oncogenic effects of calreticulin in MPNs has focused primarily on the JAK2/MPL axis, which appears to be the main oncogenic mechanism behind these diseases. However, mutant calreticulin also triggers JAK2/MPL-independent mechanisms that help to modulate the disease. These mechanisms have not been easy to analyze, either because of the powerful effects through the canonical pathway or because of the lack of experimental models that allow this analysis. In this work we have developed a *C. elegans* model, harboring MPN-like calreticulin mutations, which naturally lacks *JAK2* and *MPL* orthologs. In this model, we have observed a transcriptional alteration of genes participating in processes that have a role in cancer and MPNs, revealing that these processes can be directly perturbed by mutant calreticulin without JAK2 or MPL intervention in the worm. Interestingly, some of these processes (cellular metabolism, extracellular matrix, epigenetics, and Wnt, Hedgehog, and Notch signaling) have never been considered to be a direct consequence of calreticulin mutations without the intervention of JAK2 or MPL in humans. According to our results, the alteration of most of these processes seems to be a consequence of a loss of function of mutant calreticulin in the worm, but the dysregulation of Hedgehog signaling and *flh-3* seems to be the result of neomorphic effects of the mutant protein. Further functional analysis of this model could be useful in delineating the molecular mechanisms that induce these aberrations in nematodes, and the validation of these results in mammalian models may unravel new potential therapeutic targets in MPNs. As far as we know, this is the first time that a *C. elegans* strain with patient-like mutations is proposed as a potential model for leukemia research.

## Data Availability

The datasets generated during the current study are available in the GEO repository under accession number GSE201599.

## Supplementary Materials

The following supporting information can be downloaded at: https://www.mdpi.com/article/10.3390/cells12010186/s1, Figure S1: Comparison of length, body size, and half-moon size of WT, CRT-1(mut1/2) and CRT-1(KO) nematodes at the time of RNA extraction; Figure S2: Comparison of qPCR and microarray results; Figure S3: Heatmaps of microarray data showing DEGs that are MYC and E2F targets in CRT-1(mut1) and CRT-1(mut2) worms vs. WT or CRT-1(KO) nematodes; Figure S4: Relative expression levels of genes involved in a neomorphic function of type 1 and type 2-like mutant calreticulin in worms according to qPCR; Table S1: Summary of results obtained in the bioinformatic analyses of mutant forms of CALR and CRT-1; Table S2: Primers and probes used for qPCR analysis; Table S3: Results of microarray analysis when comparing CRT-1(mut1) vs. WT; Table S4: Results of microarray analysis when comparing CRT-1(mut2) vs. WT; Table S5: Results of microarray analysis when comparing CRT-1(mut1) vs. CRT-1(KO); Table S6: Results of microarray analysis when comparing CRT-1(mut2) vs. CRT-1(KO); Table S7: Remarkable biological processes and hallmarks found altered in both type 1 and type 2-like calreticulin-mutated nematodes and their comparison to a calreticulin-KO strain; Table S8: Results of microarray analysis when comparing CRT-1(KO) vs. CRT-1(WT).
