# Supplementary material for: Transcriptomic Analysis Reveals JAK2/MPL-Independent Effects of Calreticulin Mutations in a C. elegans Model
Source: Cells. 2023 Jan 2;12(1):186. doi: 10.3390/cells12010186 (PMC9818371; doi:10.3390/cells12010186)
Supplement: Supplementary file 1 [file cells-12-00186-s001.zip › Figures S1, S2, S3 and S4.pdf]

Supplementary Material

Transcriptomic Analysis Reveals JAK2/MPL-Independent Effects of Calreticulin Mutations in a *C. elegans* Model

Ana Guijarro-Hernández [1], Laura Eder-Azanza [1], Cristina Hurtado [1], David Navarro-Herrera [1], Begoña Ezcurra [2], Francisco Javier Novo [1,3], Juan Cabello [2] and José Luis Vizmanos [1,*]

[1] Department of Biochemistry and Genetics, School of Sciences, University of Navarra, Pamplona, Spain
[2] Center for Biomedical Research of La Rioja (CIBIR), Logroño, Spain
[3] Navarra Institute for Health Research (IdiSNA), Pamplona, Spain
* Correspondence: jlvizmanos@unav.es

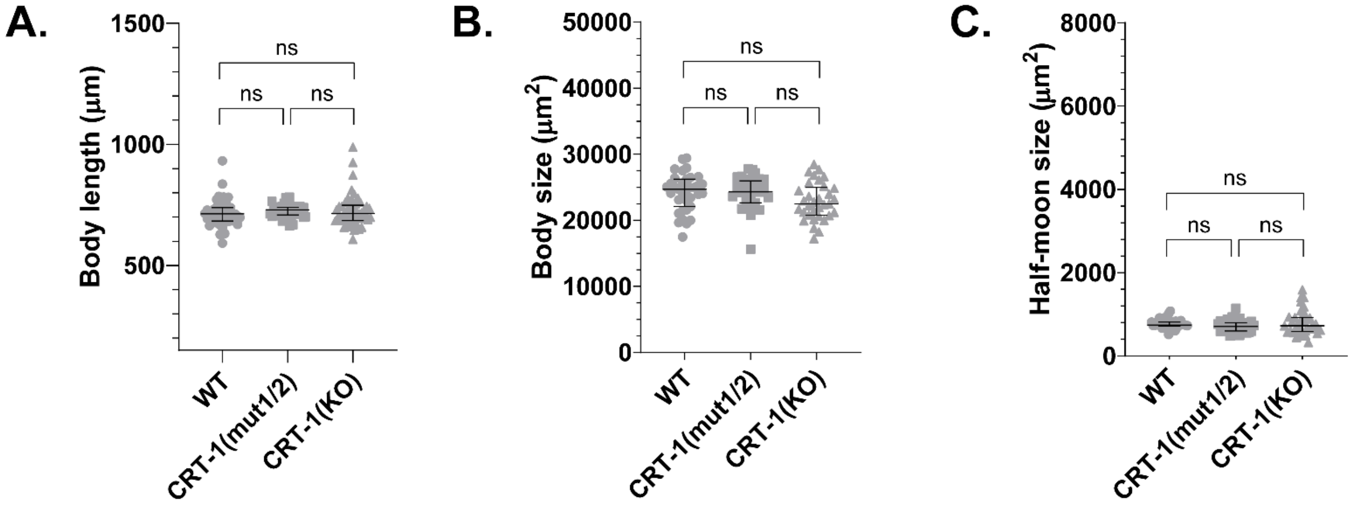

Figure S1. Comparison of length, body size and half-moon size of WT, CRT-1(mut1/2) and CRT-1(KO) nematodes at the time of RNA extraction. (**A**). Body length values of 50 worms per strain. (**B**). Body size values of 35 worms per strain. (**C**). Half-moon size values of 35 worms per strain. Individual values, median and interquartile range are represented. WT, CRT-1(mut1/2) and CRT-1(KO) worms were collected for RNA extraction at 44, 51, and 56 hours, respectively. The three plots indicate no differences among the four strains at those hours. Additionally, the length and body size of worms correspond to the values expected for L4 nematodes and the presence of the half-moon confirms that all the strains are at the L4 stage at the time of RNA extraction.

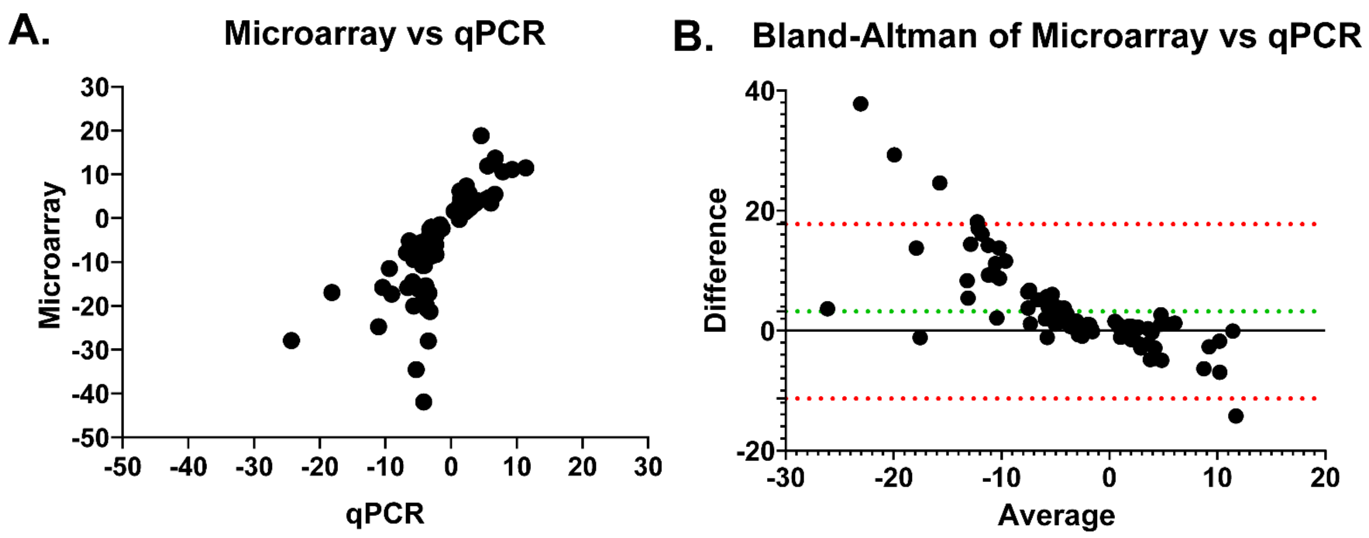

Figure S2. Comparison of qPCR and microarray results. (**A**). Representation of qPCR and microarray mean fold-change values of type 1 and type 2-like calreticulin-mutated samples vs. the mean value for WT nematodes or *crt-1* null mutants. Values for qPCR data were obtained from two independent experiments (**B**). Bland-Altman plot and analysis. The dotted horizontal green line shows the mean of the differences (bias) between the two methods, and the dotted red horizontal lines show the upper and lower limits of the 95% confidence interval for the average difference (bias ± 1.96 SD). The plot indicates a high agreement among qPCR and microarray techniques.

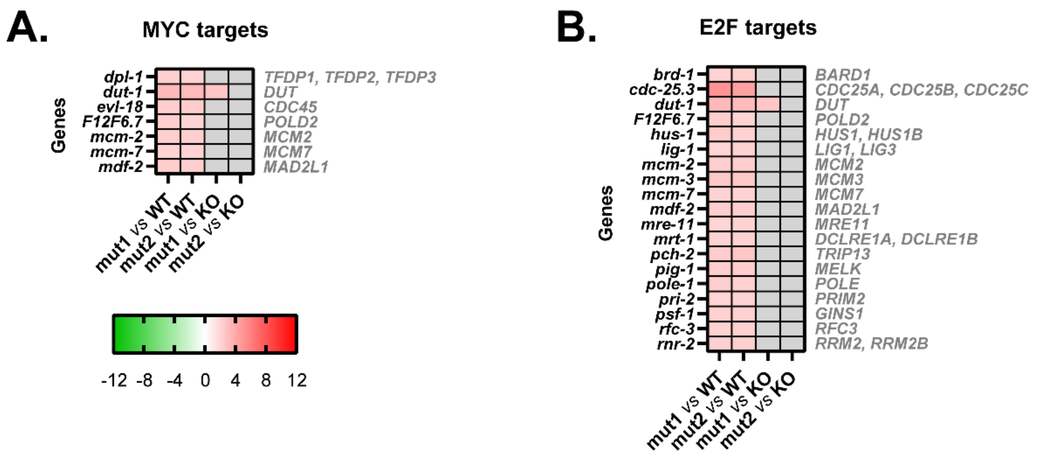

Figure S3. Heatmaps of microarray data showing DEGs that are MYC and E2F targets in CRT-1(mut1) and CRT-1(mut2) worms vs. WT or CRT-1(KO) nematodes. (**A**). MYC targets. (**B**). E2F targets. Mean fold change values are represented, ranging from shades of green (negative fold change) to shades of red (positive fold change). Non-statistically significant differences are shown in gray. Names in gray on the right are the human orthologous genes according to Ortholist2 [11].

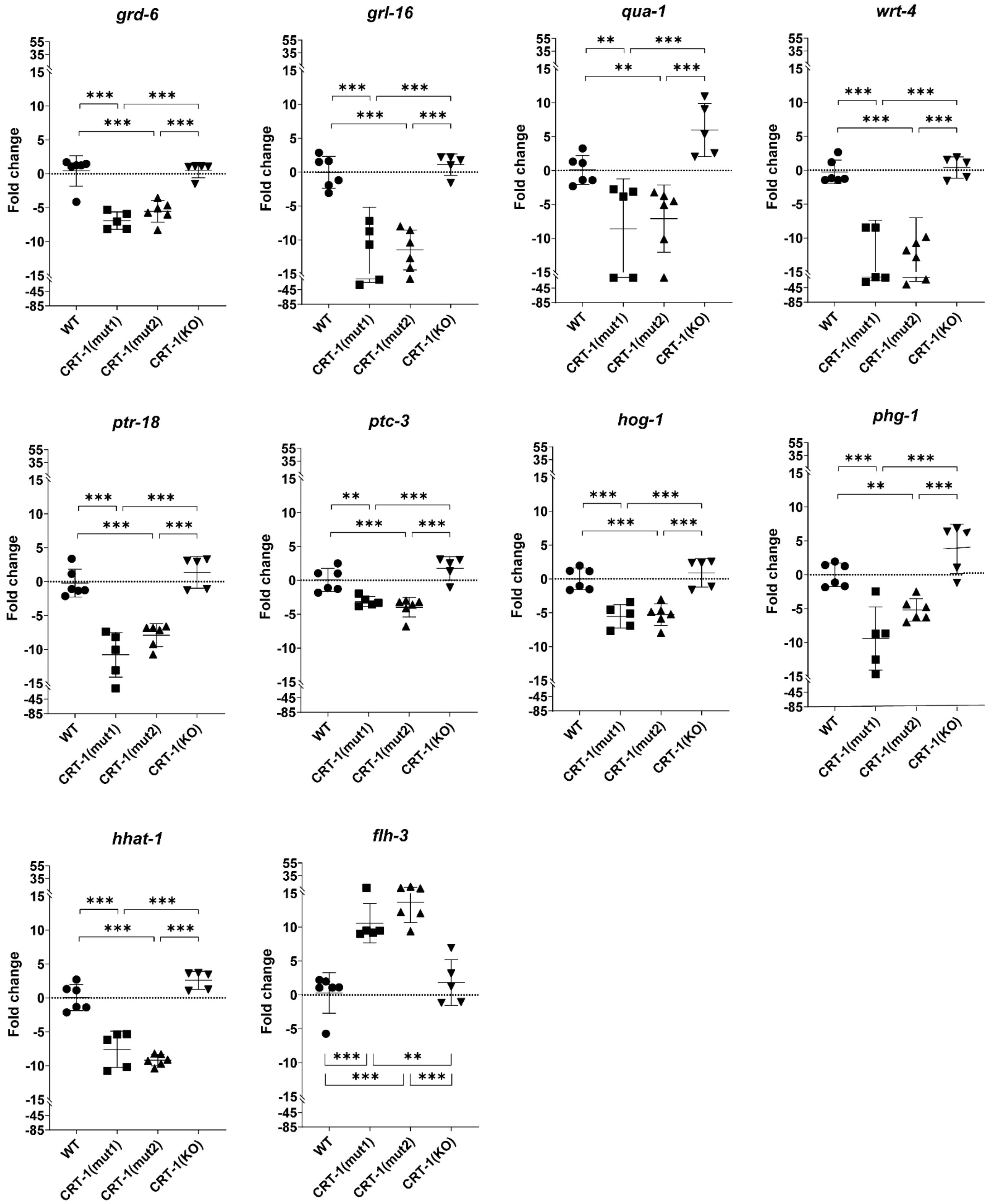

Figure S4. Relative expression levels of genes involved in a neomorphic function of type 1 and type 2-like mutant calreticulin in worms according to qPCR. The expression of these genes is opposite between CRT-1(mut1)/CRT-1(mut2) worms and the CRT-1(KO) strain. Most of them participate in Hedgehog signaling, except for *flh-3*, which is involved in negative regulation of transcription. Individual values, means and SDs are represented. Differences were considered as very significant (**) when $p < 0.01$ and highly significant (***) when $p < 0.001$.
